# ZnO:Ga-graded ITO electrodes to control interface between PCBM and ITO in planar perovskite solar cells

**DOI:** 10.1080/14686996.2019.1599695

**Published:** 2019-04-25

**Authors:** Hae-Jun Seok, Azmat Ali, Jung Hwa Seo, Hyun Hwi Lee, Na-Eun Jung, Yeonjin Yi, Han-Ki Kim

**Affiliations:** a School of Advanced Materials Science and Engineering, Sungkyunkwan University, Suwon, Republic of Korea; b Department of Materials Physics, Dong-A University, Busan, Republic of Korea; c Pohang Accelerator Laboratory, POSTECH, Pohang, Republic of Korea; d Institute of Physics and Applied Physics, Yonsei University, Seoul, Republic of Korea

**Keywords:** Ga-doped ZnO, ITO, electron extraction layer (EEL), perovskite solar cells, graded sputtering, 50 Energy Materials, 107 Glass and ceramic materials; 201 Electronics / Semiconductor / TCOs, 209 Solar cell / Photovoltaics, 306 Thin film / Coatings

## Abstract

Ga-doped ZnO (GZO)-graded layer, facilitating electron extraction from electron transport layer, was integrated on the surface of transparent indium tin oxide (ITO) cathode by using graded sputtering technique to improve the performance of planar n-i-p perovskite solar cells (PSCs). The thickness of graded GZO layer was controlled to optimize GZO–indium tin oxide (ITO) combined electrode for planar n-i-p PSCs. At optimized graded thickness of 15 nm, the GZO–ITO combined electrode showed an optical transmittance of 95%, a resistivity of 2.3 × 10^−4^ Ohm cm, a sheet resistance of 15.6 Ohm/square, and work function of 4.23 eV, which is well matched with the 4.0-eV lowest unoccupied molecular orbital of [6,6]-phenyl-C_61_-butyric acid methyl ester. Owing to enhanced extraction of electron by the graded GZO, the n-i-p PSC with GZO–ITO combined electrode showed higher power conversion efficiency (PCE) of 9.67% than the PCE (5.25%) of PSC with only ITO electrode without GZO-graded layer. In addition, the GZO integrated-ITO electrode acts as transparent electrode and electron extraction layer simultaneously due to graded mixing of the GZO at the surface region of ITO electrode.

## Graphical abstract

**Figure:**
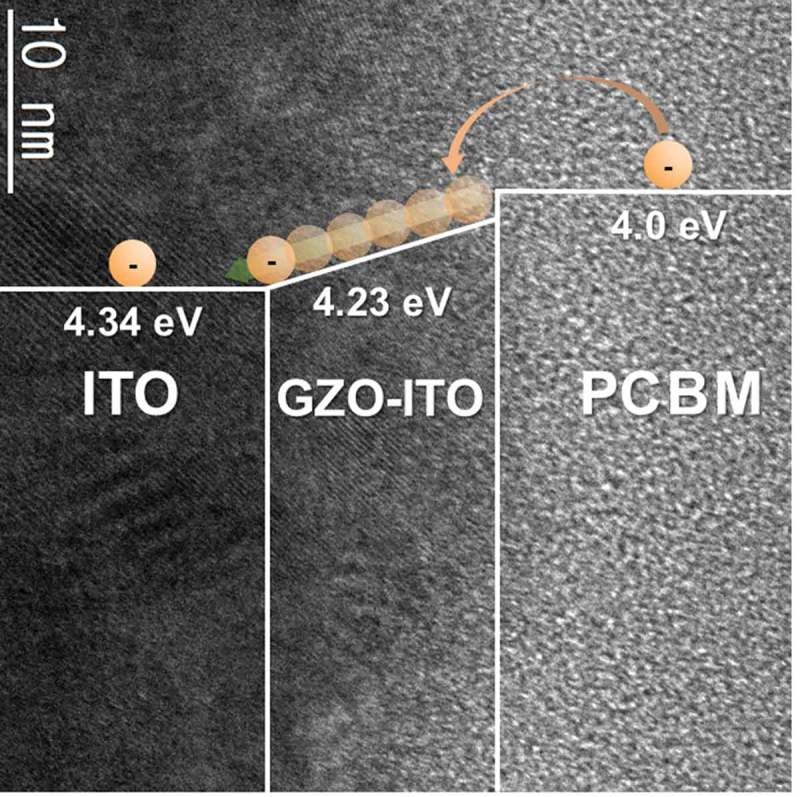

## 1. Introduction

Cost-effective photovoltaic devices, such as dye-sensitized, organic, and quantum dot solar cells, have been intensively explored aiming toward lowering device fabrication costs and enhancing power conversion efficiency (PCE) to the values comparable to or higher than those of typical Si-based thin-film photovoltaics [1–4]. Such PCEs have not been achieved yet due to imperfect, primarily owing to imperfect device fabrication, instability of organic layers, low light absorbing efficiency, and unexpected charge recombination. As another promising candidate substituting Si-based thin-film photovoltaics, organic–inorganic hybrid lead halide perovskite solar cells (PSCs) have recently attracted wide interest in photovoltaic applications because of their impressive PCEs exceeding 23.3% and simple device structure [5–12]. Importantly, the perovskite thin-film absorber can be deposited using low-cost printing and abundant starting materials, which is beneficial for the preparation of inexpensive photovoltaic devices as well as flexible photovoltaics. High PCEs are the result of a very high-absorption coefficient and mobilities of the photogenerated electrons and holes of the hybrid perovskites [13–17]. Since the PCEs of PSCs are largely determined by the exciton formation efficiency in the perovskite active layer and efficiency of carrier extraction through buffer layer, most research on increasing PCE has been focused on the synthesis of perovskite active layers and interfacial layers. However, transparent electrodes also strongly affect PCEs of PSCs because electron-hole generation in active layer and extraction efficiency in interfacial region are intimately related to optical transparency and electrical resistivity of the transparent electrodes. Carrier extraction through electrodes also significantly influences the PCE of PSCs because the perovskite absorber layer is usually sandwiched between cathode and anode electrodes [18–22]. Typically, most of the PSCs have been fabricated on chemical vapor deposited F-doped SnO_2_ or physical vapor deposited Sn-doped In_2_O_3_ electrode depending on their fabrication process or planar (n-i-p or p-i-n) structures [23–28]. In particular, sputtered ITO films have been mainly used as transparent electrode for p-i-n structure PSCs. However, the work function of the bare ITO is not well matched with PBCM which is well-known electron transport layer (ETL) in organic solar cells and PSCs. Therefore, it is necessary to improve the electron extraction by matching the energy level between [6,6]-phenyl-C_61_-butyric acid methyl ester (PCBM) and ITO. In general, un-doped ZnO films have poor electrical properties due to the low carrier concentration. Doping ZnO with appropriate III-group impurities (Al, Ga, or In) can increase the electrical conductivity and optical properties of ZnO thin films [29–31]. Al-, Ga-, and In-doped ZnO (AZO, GZO, and IZO) films are transparent to most of the solar spectrum used for photovoltaic solar cells, and their sheet resistances are comparable to those of ITO films. Among these materials, GZO is cheaper than IZO and more stable than AZO (more resistant to oxidation). Furthermore, the covalent bond length of Ga–O (1.92 Å) is similar to that of Zn–O (1.97 Å). Because of this small difference, only a small deformation of the ZnO lattice occurs, resulting in higher conductivity. In addition, the GZO has an appropriate work function (4.4 eV) as an electron extraction layer (EEL) similar to ITO work function (4.4–4.5 eV) [32,33]. Therefore, the GZO embedded in the surface of ITO electrode can control the surface of the ITO electrode and act as an effective electron extraction between ITO and PCBM layer [34,35]. For those reasons, ZnO-based ETL has been employed in organic photovoltaics [36].

Herein, we developed a GZO-graded ITO electrode through a co-sputtering process to improve the electron extraction properties. By graded sputtering of GZO on the surface of the ITO electrode, we fabricated GZO-graded ITO electrodes which allow us to control the interface and work function of ITO electrode. By comparing electrical and optical properties, we determined optimal-graded GZO layer thickness on the ITO electrode. In addition, we compared the performance of PSCs with ITO monolayer, GZO/ITO bilayer, and GZO-graded ITO electrode to show potential of GZO-graded ITO electrode as effective EEL. Based on energy band structure and interfacial structure studies, we proposed the possible electron extraction mechanism of GZO-graded ITO electrodes in operation of PSCs.

## 2. Experimental details

### 2.1. Graded sputtering of GZO onto ITO

**Figure 1.**
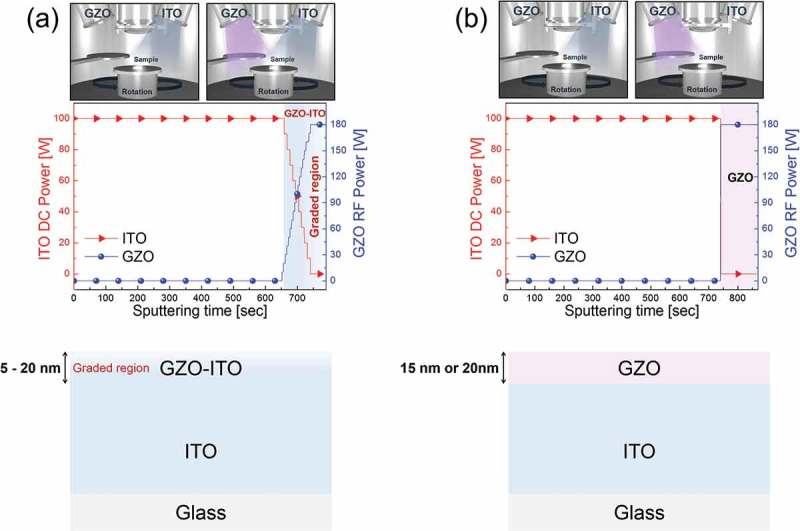
Power profile applied to ITO and GZO target during graded sputtering as a function of sputtering time and cross-sectional structure of (a) GZO-graded ITO electrode and (b) GZO/ITO bilayer electrode fabricated by DC and RF co-sputtering process. Top panels demonstrate DC and RF power applied to ITO and GZO target to fabricate GZO-graded ITO electrode and GZO/ITO bilayer electrode for planar n-i-p PSCs.

Graded GZO layer was embedded onto ITO by DC and RF magnetron co-sputtering with 15°-tiled multi-cathode guns at the room temperature. The glass substrates were first cleaned with standard cleaning procedures (acetone-isopropyl alcohol-methanol) and then rinsed in deionized water. After loading cleaned glass substrates from the load lock chamber to process chamber, ITO film was deposited by applying a constant DC power of 100 W, Ar flow rate of 20 sccm, and a working pressure of 3 mTorr using a 4-in ITO target (Dasom RMS). As shown in Figure 1(a), at the end of sputtering of ITO electrode, the GZO and ITO co-sputtering was started by applying RF power to the GZO target. The DC power applied to ITO target was reduced from 100 to 0 W while RF power applied to GZO target was increased from 0 to 180 W, simultaneously for 88 s as shown in power profile in Figure 1(a). The GZO-graded region thickness on ITO electrode was fixed at 5, 10, 15, and 20 nm as illustrated in the bottom panel of Figure 1(a). Figure S1 shows the DC and RF power profiles applied to the ITO and GZO targets during graded sputtering for controlling of the thickness of the GZO-graded layer as a function of the sputtering time. The total thickness of GZO-graded ITO film was fixed at 150 nm. For comparison, we prepared GZO/ITO bilayer electrode with 15 and 20-nm-thick GZO ETL using two-step sputtering as shown in Figure 1(b). During the bilayer sputtering or graded sputtering, the glass substrate rotating constantly at 30 rpm improves film uniformity. After sputtering of the GZO-graded ITO electrode and the GZO/ITO bilayer electrode, all samples were rapidly thermally annealed at a temperature of 600 °C for 10 min to improve their electrical and optical properties of electrodes [37,38].

### 2.2. Characterization of GZO-graded ITO electrode

Through Hall measurements (HL5500PC, Accent Optical Technology, UK) and UV/visible spectral analysis (UV 540 spectrometer, Unicam, Japan), the electrical and optical properties of the GZO-graded ITO, GZO/ITO bilayer, and ITO monolayer cathode were examined. The X-ray photoelectron spectroscopy (XPS) depth profile and survey analysis of the optimized GZO-graded ITO cathode were carried out to investigate composition of graded region (UV 540 spectrometer, Unicam, Japan). The crystallinity of GZO-graded ITO cathode was investigated by grazing incident X-ray diffraction measurement (GID; PLS-II, Korea). The incident angle was set to 0.1° to enhance surface region, which is smaller than a critical angle (or total external reflection angle) of ITO film (~0.155°). The GID measurement was performed with X-rays of 0.06925 nm wavelength (*λ*) at 5 A materials science beam line of the Pohang Light Source II (PLS-II) in Korea. In addition, the surface morphology of the ITO, GZO, and GZO-graded ITO electrodes was observed by field emission scanning electron microscopy (FESEM; JSM-7600F, JEOL, Japan). The work function of the ITO, GZO, and GZO-graded ITO cathodes was measured by ultraviolet photoelectron spectroscopy (UPS; ESCALB-250Xi, Thermo Fisher Scientific, USA). High-resolution transmission electron microscopy (HRTEM: JEM-2100F – 200 keV; JEOL, Japan) analysis was employed to investigate the microstructure and interface region of the optimized GZO-graded ITO electrode used in the PSCs.

### 2.3. Fabrications and evaluations of the planar-type n-i-p PSCs

To demonstrate the feasibility of a GZO-graded ITO cathode, PSCs were fabricated using the ITO monolayer, GZO/ITO bilayers, and GZO-graded ITO cathodes. The ITO monolayer, GZO/ITO bilayers, and GZO-graded ITO cathodes were cleaned using deionized water, acetone, isopropanol, and ethanol separately in ultrasonic bath for 15 min each to remove the organic and inorganic residues if it had any. The transparent cathodes underwent UV/ozone treatment for 10 min before the deposition of a PCBM layer. Then, an ETL, the PCBM (25 mg/ml in chlorobenzene), layer was fabricated by spin-coating at 3000 rpm for 30 s in a glove box filled with N_2_ and the films were annealed at 80 °C for 5 min. Subsequently, a perovskite precursor of 45 wt% was prepared by mixing PbI_2_ and MAI with the molar ratio of 1:1 in DMF:DMSO with 7:3 which was coated onto the PCBM layer with consecutive two-step spin coating; in second step, 45 µl anhydrous chlorobenzene was dropped on the center of substrate. The films were annealed at 90 °C for 10 min to remove the solvent. As a hole transport layer, 2,2′,7,7′-tetrakis(*N,N*-di-*p*-methoxyphenyl-amine)9,9′-spirobifluorene (spiro-OMeTAD) was grown on the perovskite active layer by spin coating at 4000 rpm for 30 s and the films were annealed at 60 °C for 10 min. Finally, 6.7 nm MoO_3_ and 100 nm Ag anodes were formed successively, with a thermal evaporator by using a dumbbell-shaped metal shadow mask having an active area of 0.12 m^2^. The J–V curves were measured using simulated AM 1.5 G irradiation (100 mW/cm^2^), which was calibrated with a standard silicon photodiode under ambient condition.

## 3. Results and discussion

**Figure 2.**
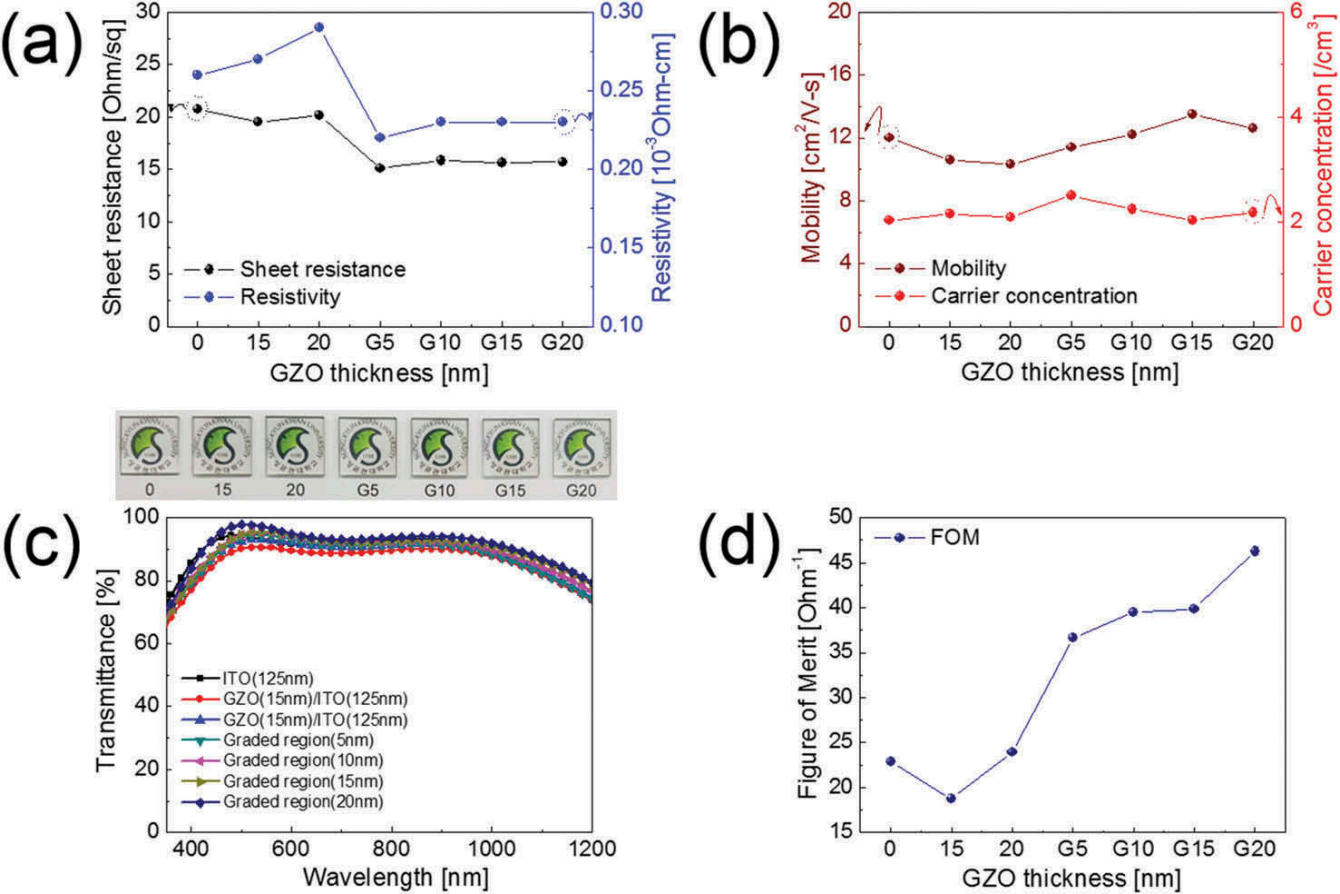
Hall measurement results of ITO monolayer, GZO/ITO bilayer, and GZO-graded ITO cathode with different graded thickness; *G5, G10, G15*, and *G20* indicate the thickness of graded layer from 5 to 20 nm. (a) Sheet resistance, resistivity, (b) carrier concentration, and mobility of the ITO monolayer (*0*), GZO/ITO bilayer (*15, 20*), and GZO-graded ITO (*G5, G10, G15, G20*). (c) Optical transmittance and (d) figure of merit (FoM = *T*^10^/*R*_sh_) of ITO monolayer, GZO/ITO bilayer, and GZO-graded ITO cathodes.

Figure 2(a) and (b) shows the Hall measurement results obtained from ITO monolayer (0), GZO/ITO bilayer (15 and 20 nm), and GZO-graded ITO cathodes with different graded thickness. For simplicity, we expressed the GZO-graded ITO with *N*-nm-graded thickness as *GN* (*G5* means the GZO-graded ITO with 5-nm-thick graded region), here after. Before fabrication of GZO-graded ITO and GZO/ITO bilayer, we investigated the electrical and optical properties. Compared to 125-nm-thick ITO monolayer and GZO/ITO bilayer, the GZO-graded ITO cathode showed a lower sheet resistance and resistivity. Because the resistivity was mainly affected by the quality of bottom ITO electrode, the GZO-grade ITO cathodes exhibited a similar low resistivity and sheet resistivity regardless of graded GZO layer thickness. Lower resistivity of GZO-graded ITO films is closely related to the somewhat higher carrier mobility than GZO/ITO bilayer as shown in Figure 2(b) due to diffused interface between GZO EEL and ITO layer. The slightly lower carrier mobility of the GZO/ITO bilayer electrode could be attributed to the presence of a sharp interface between the GZO layer and ITO layer, which act as scattering sources. As a result, the 15-nm-thick GZO-graded ITO (*G15*) electrode showed that the lowest sheet resistance is 15.6 Ohm/square and the highest mobility of 13.5 cm^2^/(V s). The optical transmittance results of ITO monolayer, GZO/ITO bilayer, and GZO-graded ITO cathodes with different GZO-graded layer were shown in Figure 2(c). The GZO/ITO bilayer cathode showed slightly lower optical transmittance of 90.43% at a wavelength of 550 nm than ITO monolayer and GZO-graded ITO electrodes due to the reflection at the sharp interface between the GZO and ITO layer. Due to the slightly low optical transmittance of the GZO/ITO bilayer cathode, the exciton generation of the perovskite active layer decreased. However, GZO-graded ITO cathodes showed similar optical transmittance at a wavelength of 550 nm to ITO monolayer. Then 15-nm-thick GZO-graded ITO cathode showed the highest optical transmittance of 95% at a wavelength of 550 nm among GZO-graded ITO electrode. Since the interface between the GZO and ITO electrode disappeared, the GZO-graded ITO electrode showed high optical transmittance. Even the existence of GZO-graded layer, the GZO-graded ITO showed higher optical transmittance than ITO monolayer (92.79%) due to high optical transmittance (96.04%) of GZO single layer. The optimization of the electrical and optical properties of GZO single layer according to the power, Ar flow rate, and working pressure of the GZO single layer is shown in Figures S2–S4. In addition, the optical transmittance of optimized GZO single layer with 20-nm thickness was compared to the optical transmittance of other samples at a wavelength of 400–800 nm as shown in Figure S5. To determine optimum GZO-graded thickness, we compared figure of merit (FoM = *T*^10^/*R*_sh_) value, which were calculated from the average optical transmittance (*T*) and the sheet resistance (*R*_sh_), as shown in Figure 2(d). Table 1 summarizes the FoM values of ITO monolayer, GZO/ITO bilayer, and GZO-graded ITO cathodes. The PSC fabricated on the ITO monolayer cathode showed a PCE of 5.25%. Because the higher FoM value generally indicates the better quality of transparent electrodes, we also decided the optimum thickness of the graded GZO by calculation of FoM of the GZO-graded ITO electrodes. Based on FoM calculation, we found that the 20-nm-thick GZO-graded ITO electrode had the highest FoM value of 46.23 Ohm^−1^, which is much higher than ITO monolayer and GZO/ITO bilayer cathodes.

**Table 1.** Figure of merit (FoM = *T*^10^/*R*_sh_) values of ITO monolayer, GZO/ITO bilayer, and GZO-graded ITO cathodes calculated from an average optical transmittance (*T*) and a sheet resistance (*R*_sh_).

	0	15	20	G5	G10	G15	G20
FoM value [Ohm^−1^]	22.85	18.77	23.91	36.64	39.48	39.86	46.23

**Figure 3.**
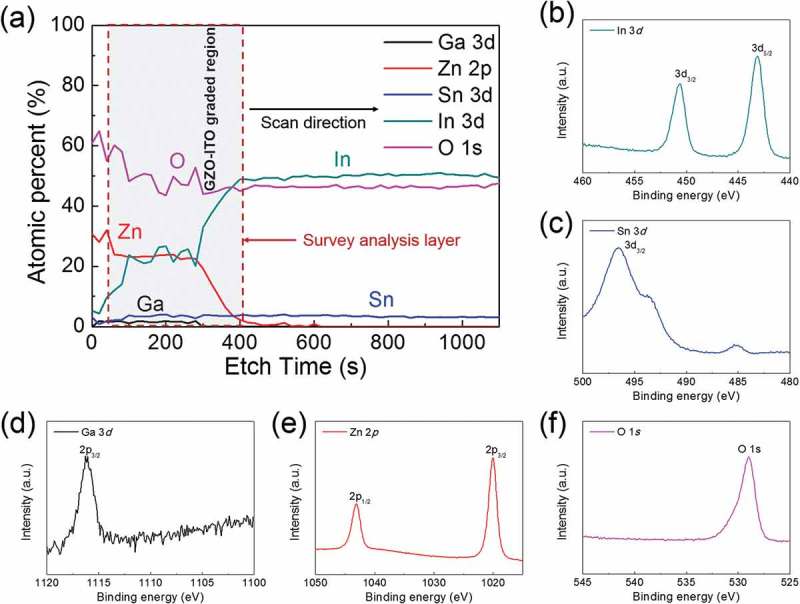
(a) XPS depth profile of 20 nm thick GZO-graded ITO cathode as a function of etch time. (b)–(f) The narrow-scan XPS spectra of In 3*d*, Sn 3*d*, Ga 3*d*, Zn 2*p*, and O 1*s* peaks obtained from the GZO-graded ITO region.

To investigate composition of the GZO–ITO-graded region, XPS depth profile was carried out. Figure 3(a) shows XPS depth profile of the FoM-based optimum GZO-graded ITO (*G20*) cathode as a function of etch time. From XPS depth profile, we clearly distinguished the bottom ITO electrode and GZO–ITO graded layer. Constant In, Sn, and O atomic percent region below GZO-graded region indicated the crystalline ITO (c-ITO) electrode with a low resistivity. Due to graded sputtering of GZO and ITO target as explained in Figure 1(a), the XPS depth profile of the GZO–ITO-graded region exhibited increasing Zn, Ga, and O atomic percentages and decreasing In and Sn atomic percentages. The sloped atomic percentage at the graded region confirmed that the surface of GZO-graded ITO cathode ended with the GZO layer, which is advantageous for extraction of electron from the PCBM layer. Through the typical XPS survey of graded region, the narrow-scan XPS spectra of In 3*d* (450.6 and 443.1 eV), Sn 3*d* (496.9 eV), Zn 2*p* (1043.1 and 1020.1 eV), Ga 3*p* (1116.3 eV), and O 1*s* (529 eV) elements were observed as shown in Figure 3(b–f). The binding energy of In 3*d*, Sn *3d*, O 1*s* peaks of ITO film and Zn 2*p*, Ga 3*d*, O 1*s* of GZO film were similar to those of the previously reported ITO and GZO films [39,40]. The binding energy of In 3*d*_5/2_ at 443.1 eV measured from graded region shown in Figure 3(b) can be attributed to the In^3+^ bonding state from In_2_O_3_ [41]. The XPS spectrum of the Sn 3*d*_5/2_ is 496.9 eV in Figure 3(c) which corresponds to the Sn^4+^ bonding state from SnO_2_. The narrow-scan XPS spectra of Ga 3*d*, Zn 2*p* and O 1*s* are depicted in Figure 3(d–f). As observed, the two main peaks denoting the binding states of Zn are assigned to Zn 2*p*_3/2_ and Zn *2p_1_*_/2_, and the binding energy of O 1*s* is positioned 529 eV in all the spectrums. The binding energies of 1020.1 eV for Zn 2p_3/2_ and 1043.1 eV for Zn 2p_1/2_ slightly shift those of Zn metal due to the binding between the Zn and O. In addition, the peak of Ga *2p_3_*_/2_ slightly shifts from the positions of metallic Ga peak, which are centered at 1117.1 eV [42]. This phenomenon is ascribed to the binding of Ga–O, indicating that Ga is successfully incorporated into the ZnO structure and acts as a dopant. As shown, the peaks of Zn and Ga are centered at 1043.1, 1020.1, and 1116.3 eV, respectively. The strong Zn, Ga, In, Sn, and O peaks from graded region indicate that the GZO was well integrated in the surface region of the ITO electrode without the interface.

**Figure 4.**
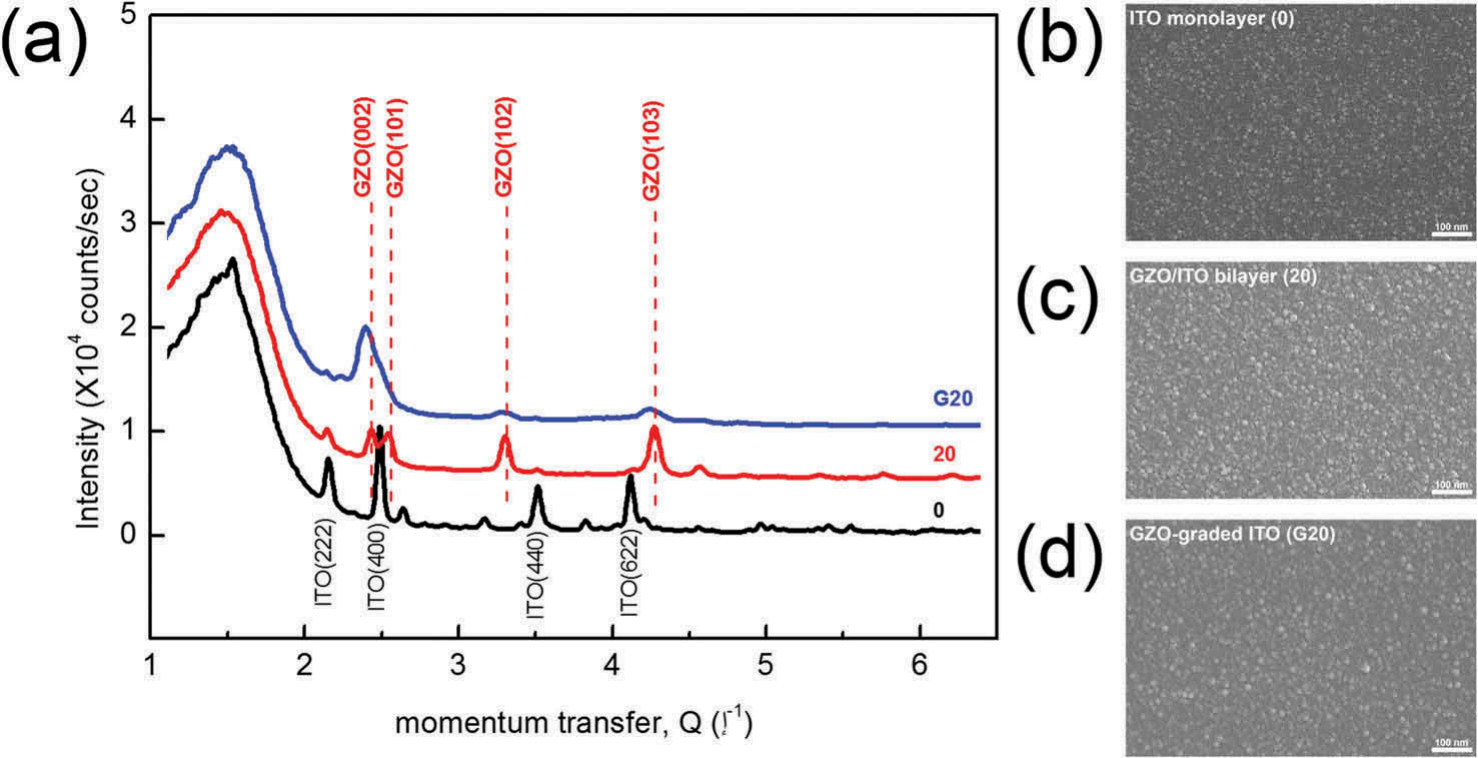
(a) Grazing incident X-ray diffraction (GID) of ITO monolayer (*0*), GZO/ITO (*20*) bilayer, and GZO-graded ITO cathode (*G20*) films. Surface FESEM images obtained from (b) ITO monolayer (*0*), (c) GZO/ITO bilayer (*20*), and (d) GZO-graded ITO cathode (*G20*).

The structure of ITO monolayer (*0*), GZO/ITO bilayer (*20*), and GZO-graded ITO (*G20*) films was investigated using GID measurement as shown in Figure 4(a) using science beamline of the PLS-II in Korea. The peaks around 2.16, 2.49, 3.52, and 4.12 Å^−1^ are all attributed to the ITO film and the peaks around 2.43, 2.55, 3.31, and 4.23 Å^−1^ correspond well to the (0 0 2), (1 0 1), (1 0 2), and (1 0 3) peaks of GZO, respectively. The GZO/ITO bilayer (*20*) film shows strong and clear wurtzite ZnO phase peaks and weak ITO peaks because X-ray penetration depth was significantly suppressed within nearly 10 nm by grazing incident condition. The GZO/ITO bilayer (*20*) film showed slightly weak crystalline surface due to existence of crystalline GZO layer (20 nm). However, there was no crystalline phase change for 20-nm-thick GZO-graded ITO (*G20*) film, but diffraction peaks became broader and weaker which may be originated from relatively poor crystallinity or effectively thinner thickness of GZO layer. Since the GZO–ITO-graded region (*G20*) was consisted of ITO (bixbyite) and GZO (wurtzite) with different crystal structure, it shows a relatively poor crystallinity. Also, the sub-grain structure of the ITO monolayer (*0*), GZO/ITO bilayer (*20*), and GZO-graded ITO (*G20*) films was confirmed by surface FESEM images in Figure 4(b–d). As expected from GID examination, the ITO monolayer (*0*) showed a complete crystalline surface after rapidly thermally annealing at a temperature of 600 ° C in Figure 4(b). However, due to existence of the GZO bilayer (20) and GZO-graded layer (*G20*), the FESEM images in Figure 4(c) and (d) showed different surface morphology. As shown in Figure 4(c), the GZO/ITO bilayer (*20*) showed slightly weak crystalline surface due to GZO single layer (20 nm) covering ITO layer. Also, the GZO-graded layer (*G20*) confirmed that the crystalline of the surface was relatively poor in Figure 4(d). Because the indium oxide (bixbyite) and zinc oxide (wurtzite) crystal structures are incompatible, crystallization is frustrated in multicomponent GZO-graded layer. In GZO–ITO mixed layer, ZnO plays an important role in stabilizing the amorphous structure via tetrahedron edge sharing like InGaZnO [43].

**Figure 5.**
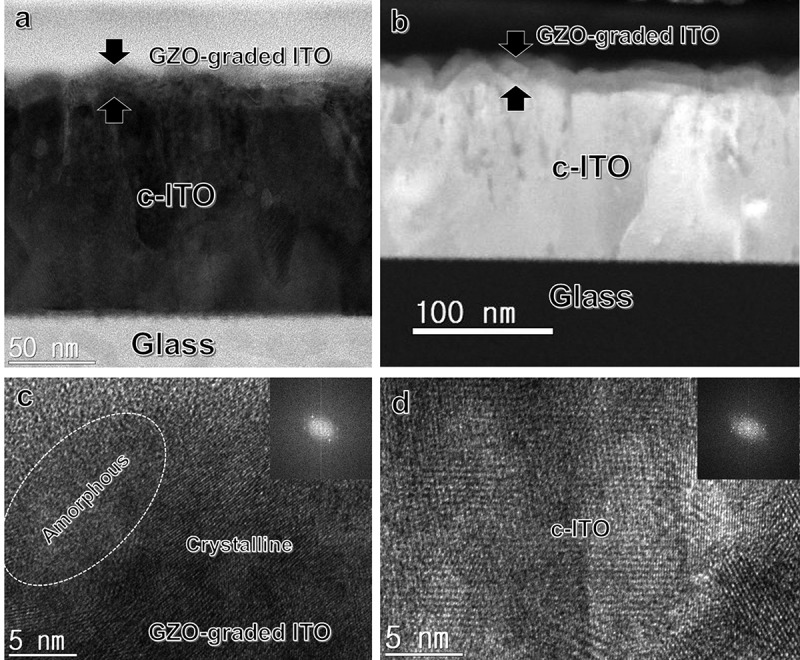
Cross-sectional HRTEM image of a GZO-graded ITO/c-ITO/glass sample (a). Bright field (BF) and (b) dark field (DF) images of the GZO-graded ITO electrode. Enlarged HRTEM image of (c) GZO-graded ITO region and (d) crystalline ITO with an inset of fast Fourier transform pattern, respectively.

To investigate the microstructure and interface structure of the GZO-graded ITO film, TEM examination was carried out. Figure 5(a) and (b) shows bright field (BF) and dark field (DF) images of the GZO-graded ITO film recorded by cross-sectional HRTEM. Based on XPS depth profile analysis, we clearly distinguished the bottom c-ITO electrode and GZO–ITO-graded layer. In HRTEM images, bottom ITO electrode showed well-developed columnar structures. In addition, due to the resputtering effect, the surface of the ITO electrode with a fairly rough surface morphology was observed [44]. Therefore, the GZO–ITO-graded layer indicated by arrows follows surface morphology of the bottom ITO electrode. Figure 5(c) shows an enlarged cross-section TEM image and fast Fourier transform (FFT) patterns obtained from GZO-graded ITO layer. As we expected from GID examination and SEM images, the GZO-graded ITO layer had both the amorphous and crystalline structure. In particular, the weak spots in the FFT pattern obtained from the GZO-graded ITO layer indicate that it shows a poor crystallinity. As shown in Figure 5(d), the c-ITO below GZO–ITO-graded region exhibited a well-grown polycrystalline bixbyite structure with the strong spots in the FFT pattern.

**Figure 6.**
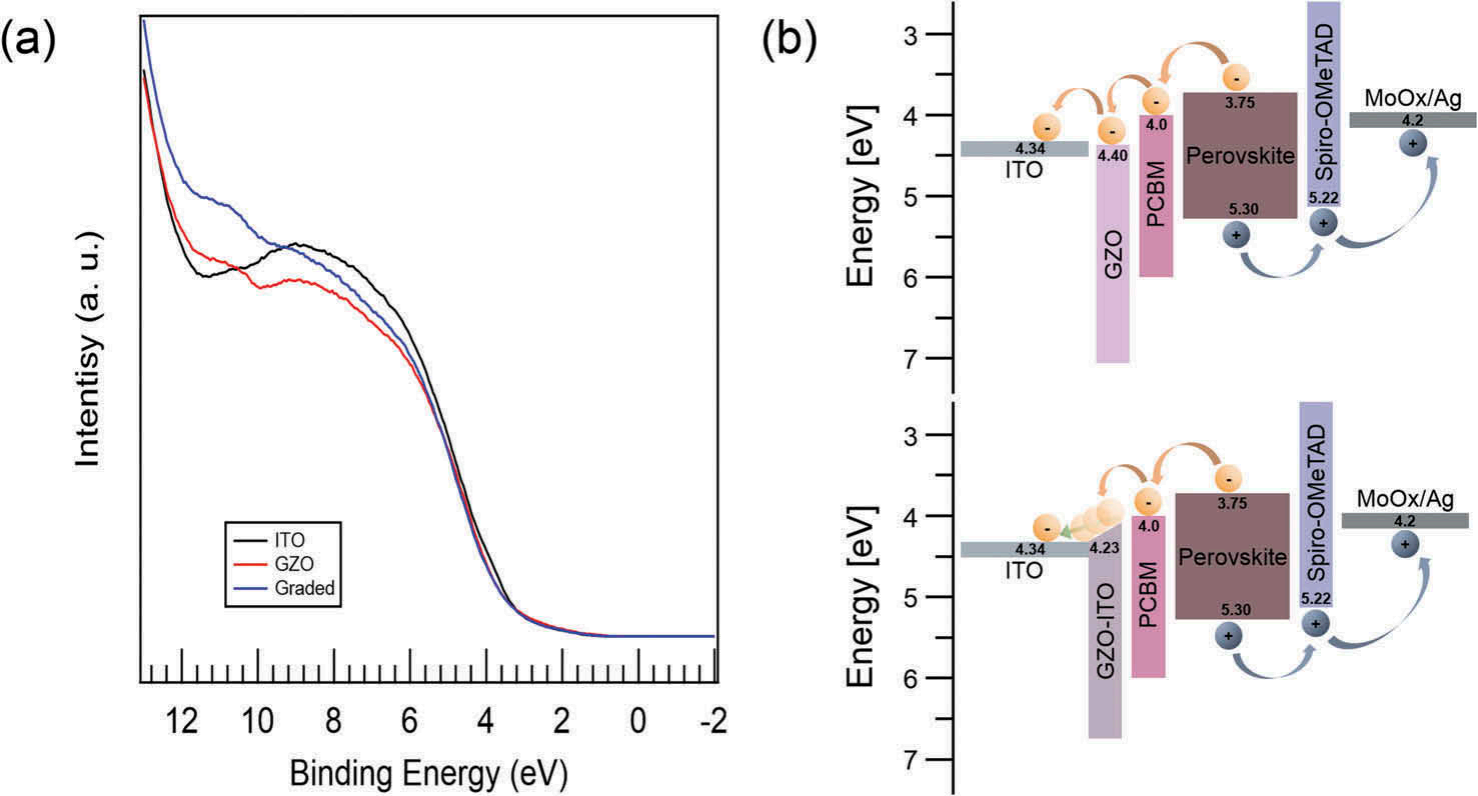
(a) UPS spectrum used to determine work function of ITO, GZO, and GZO-graded ITO film. (b) Energy band diagram of the PSCs on GZO/ITO bilayer and GZO-graded ITO cathodes.

Through UPS analysis, we measured the modified work function of GZO-graded ITO film to identify the electron extraction mechanism at the interfaces from the PCBM layer. Figure 6(a) shows spectrum used to determine work function of the ITO (125 nm), GZO (20 nm), and 15-nm-thick GZO-graded ITO film obtained from UPS analysis. The work function (Φ) of the films was defined by Φ = *hv* − Δ*E*, where *hv* is the photon energy (21.22 eV) and Δ*E* is determined from the distance of the binding energy between the secondary electron emission cutoff edge in the UPS spectra [33,45]. The work function of the GZO-graded ITO film (Φ = 4.23 eV) was lower than the ITO film work function (Φ = 4.34 eV) even the surface is modified due to the integrated GZO–ITO graded region, as shown Figure 6(a). However, the work function of the GZO film (Φ = 4.40 eV) was slightly higher than those of the ITO film as shown in Figure S6. Based on the UPS analysis, the energy band diagram illustration of respective PSCs to compare GZO/ITO bilayer and GZO-grade ITO cathode applied to PSCs is shown in Figure 6(b). The PSC with GZO-graded ITO cathode shows the improved electron extraction process at the interfaces from the PCBM layer and the ITO layer due to lower work function of GZO–ITO-graded region. Therefore, the interface controlled GZO-graded ITO cathode significantly improves solar cell performance because it effectively enhances the electron extraction, transport, and suppresses charge recombination. However, the GZO/ITO bilayer cathode, which has a work function slightly higher than that of ITO work function, exhibits stepped electron transport at the interfaces between the PCBM layer and the ITO layer, resulting in a decrease in the PCE of PSCs.

**Figure 7.**
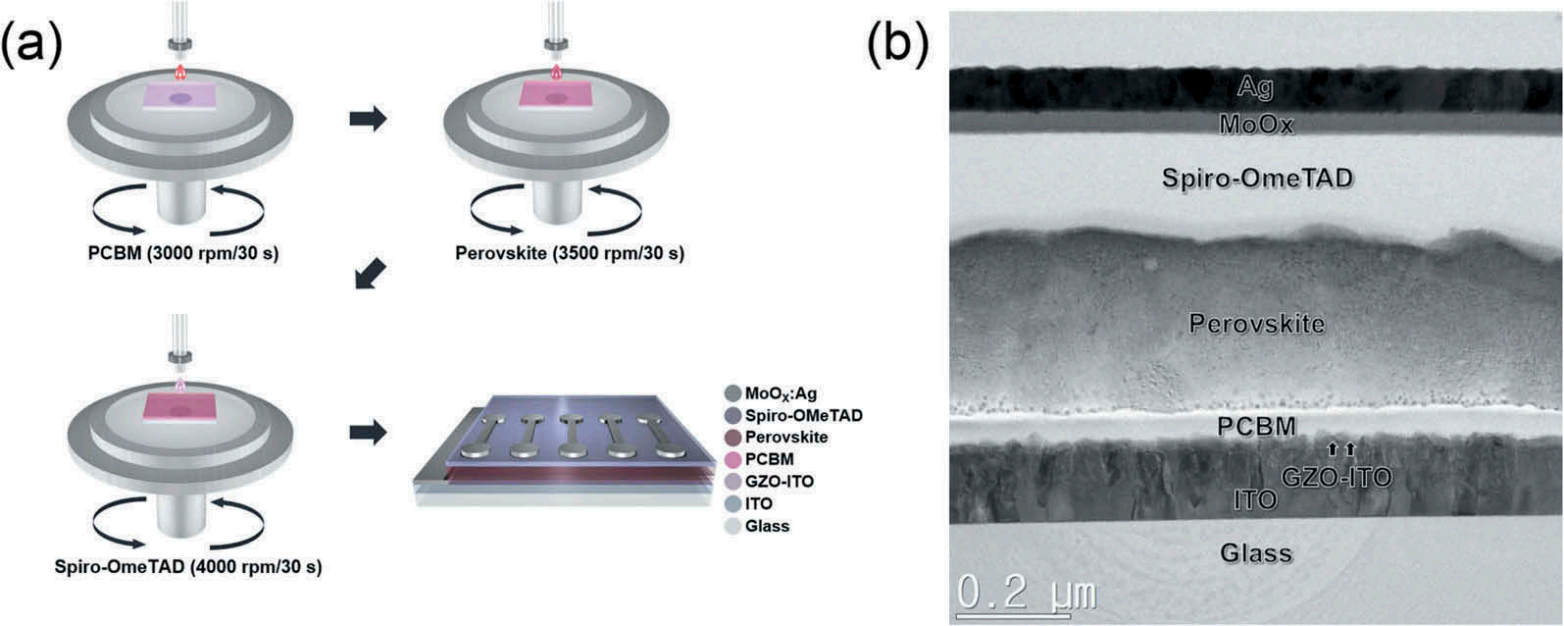
(a) Schematic of a PSC fabrication procedure on a GZO-graded ITO cathode. (b) Cross-section HRTEM image of conventional planar n-i-p PSCs fabricated using GZO-graded ITO cathode.

To use the GZO-graded ITO cathode in PSCs, we fabricated the typical planar n-i-p PSCs. Figure 7(a) shows schematic representation of the PSC fabrication processes on a GZO-graded ITO cathode by using a spin coating processes. Figure 7(b) is a cross-sectional TEM image of a PSC manufactured using GZO-graded ITO cathode with the structure glass/ITO/GZO-graded region/PCBM/perovskite/spiro-OMeTAD/MoO*_x_*:Ag. The well-distinguished interface between each layer in the cross-sectional TEM image shows evidence that the interface reaction did not occur. Also, the existence of GZO-graded region indicated by the arrow was confirmed. Through this GZO-graded region, electron was effectively extracted from PCBM layer to transparent ITO cathode layer.

**Figure 8.**
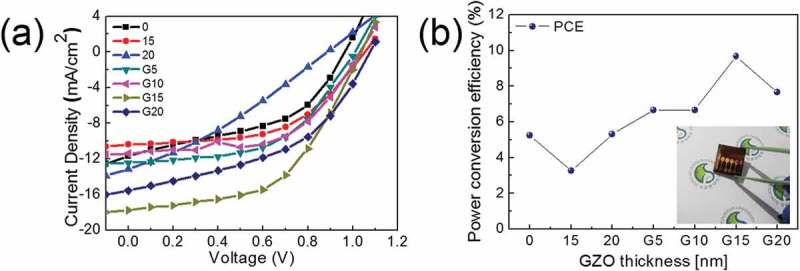
(a) Current density–voltage (J–V) curves (b) PCEs of PSCs fabricated on the ITO monolayer (*0*), GZO/ITO bilayer (*15, 20*), and GZO-graded ITO cathodes under AM 1.5 G solar light. The insert in (b) showed a PSC photograph fabricated on GZO-graded ITO cathode (*G15*).

Figure 8 depicts current density–voltage (J–V) curves and PCE of the PSCs on ITO monolayer, GZO/ITO bilayer, and GZO-graded ITO cathodes as according to the top GZO and GZO-graded layer thickness. Table 2 summarizes the key J–V parameters of the n-i-p PSCs. The PSC fabricated on the ITO monolayer cathode showed a PCE of 5.25%. However, the PCEs (3.26% and 5.31%) of the PSCs based on the GZO/ITO bilayer cathode at the GZO layer thickness 15 and 20 nm were slightly lower than or similar to the PCE of the PSC based on ITO single layer cathode. The reason for the decrease in PCE is due to the low transmittance and electron mobility caused by the sharp interface between the GZO and ITO layer. Also, due to scattering at the interface, GZO/ITO bilayer cathodes led to lower short-circuit current densities (*J*_SC_) and fill factors (FFs). The high work function of GZO layer prevented effective electron extraction from the PCBM layer as illustrated in Figure 6(b), and hence the bilayer-based PSCs showed lower PCEs. However, insertion of GZO-graded layer on ITO electrode improved the performance of PSCs. Regardless of GZO-graded layer thickness, the PSCs on GZO-graded ITO electrode showed higher PCEs than PSCs with the ITO monolayer and GZO/ITO bilayer cathodes, because all key parameters of J–V curves are significantly improved compared to ITO monolayer and GZO/ITO bilayer cathodes. In particular, for a GZO-graded ITO cathode with the GZO-graded layer thickness of 15 nm, the J–V curve of the highest-performing device yields a *V*_OC_ of 1.03 V, a *J*_SC_ of 17.76 mA cm^2^, a FF of 52.0%, and a PCE of 9.67%. Although the FoM value of *20G* is higher than *15G*, the *15G*-based PSC showed a higher PCE than *20G*-based PSC. High electron mobility and electrical conductivity contribute to large *J*_SC_ and FF values and the enhanced electron extraction and reduced charge recombination affect high *V*_OC_ values. These results can still demonstrate that the GZO-graded ITO cathode is suitable for conventional planar n-i-p PSCs.

**Table 2.** Key parameters of champion PSCs based on ITO monolayer, GZO/ITO bilayer, and GZO-graded ITO cathode.

Parameter	*V*_OC_ [V]	*J*_SC_ [mA/cm^2^]	FF [%]	PCE [%]
0	0.97	11.67	46.4	5.25
15	0.97	11.82	28.4	3.26
20	1.01	13.00	40.5	5.31
G5	1.01	12.43	53.0	6.66
G10	1.03	11.41	56.5	6.66
G15	1.03	17.76	52.0	9.67
G20	1.07	15.54	45.0	7.65

**Figure 9.**
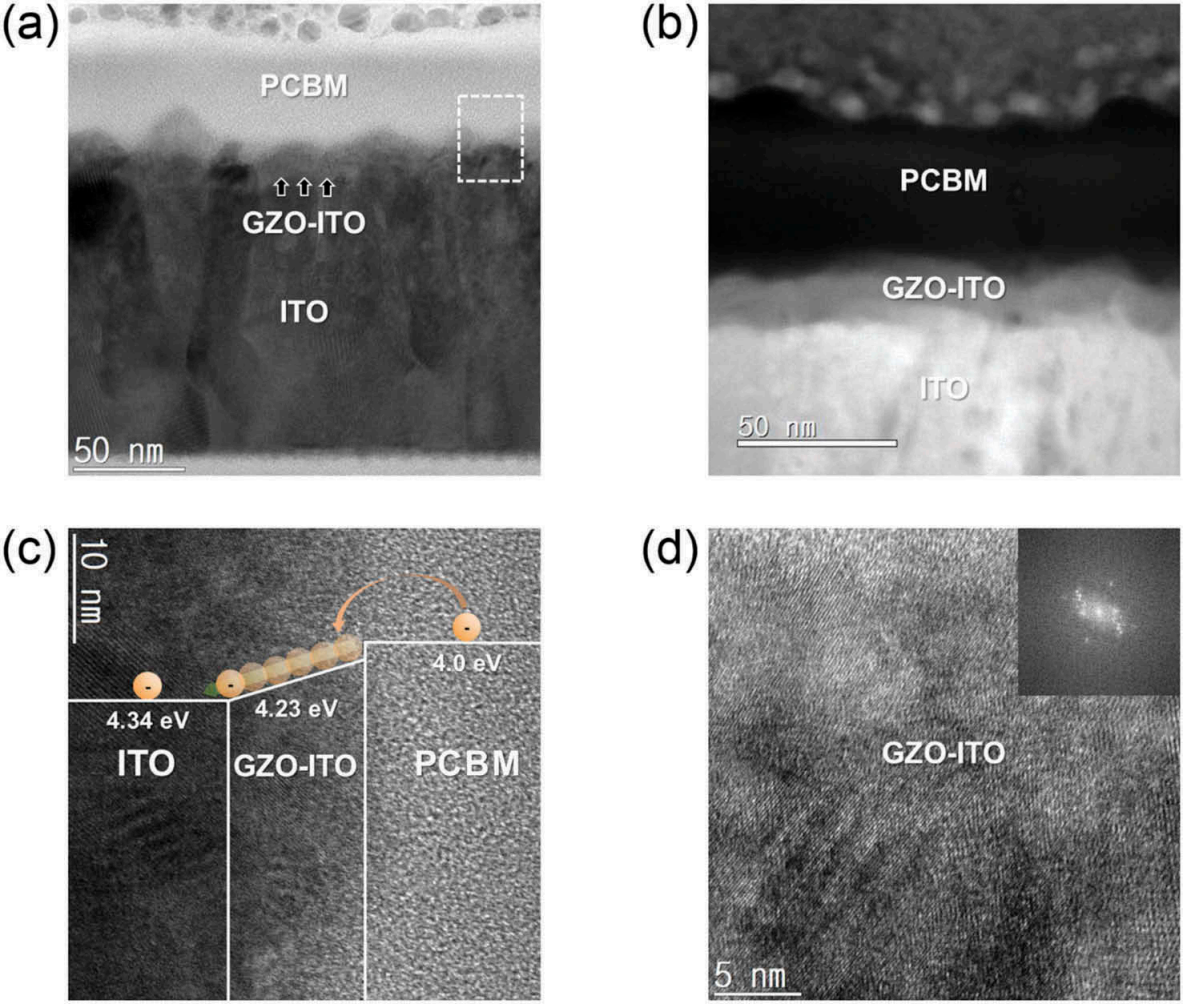
Enlarged cross-sectional TEM (a) bright field and (b) dark field images of GZO-graded ITO cathode. (c) Electron extraction mechanism through the GZO-graded ITO region between PCBM and ITO cathode. (d) Enlarged HRTEM image of GZO-graded ITO region with an inset of fast Fourier transform pattern.

Based on device performance and UPS analysis results, we can suggest a possible mechanism to explain the GZO-grade ITO electrode like below. Figure 9(a) and (b) shows the BF and DF-enlarged cross-sectional TEM images of the clear interface between ITO/GZO-graded region/PCBM to demonstrate the electron extraction mechanism. The square dotted outline enlarged image in Figure 9(a) shows a schematic representation of the electron extraction mechanism from the PCBM layer to the ITO layer as shown in Figure 9(c). In addition, Figure 9(d) shows an enlarged cross-section TEM image and FFT patterns obtained from GZO-graded ITO layer. Through the weak spots of the FFT pattern, the GZO-graded ITO layer had both the nanocrystalline embedded amorphous structure. The enhanced electron extraction mechanism from the PCBM layer to the ITO layer, which are electrons extracted from the perovskite active layer, is describe below. When solar light is irradiated, excitons are generated in the perovskite active layer, which increases the generation of excitons due to the high transparency of the GZO-graded ITO cathodes. Thus, electrons generated by exciton separation move to the ITO layer through the PCBM layer. Due to the well-matched work function of the PCBM layer and GZO–ITO layer, a large number of electron carriers flowed smoothly into the conductive ITO layer with low sheet resistance before recombination takes place, as shown in Figures 6(b) and 9(c). Therefore, the GZO-graded ITO cathodes with a low sheet resistance have a high FF value. However, for PSCs with ITO monolayer cathode, the work function between the ITO (4.34 eV) and PCBM (4.0 eV) layer was significantly different, resulting in high Schottky barrier height (SBH) [46]. Therefore, extraction of electrons from PCBM, which is the ETL, is difficult to be efficient. In the case of GZO-graded ITO cathode, the work function (4.23 eV) was higher than work function (4.0 eV) of ITO monolayer cathode due to the modified surface. Therefore, electrons could be more easily extracted from the PCBM to GZO-graded layer because SBH between the PCBM and GZO-graded layer was negligible. As a result, the GZO-graded ITO cathode developed in this study is expected to be a useful transparent electrode for PSCs owing to the well-matched work function, low sheet resistance, and high optical transmittance.

## 4. Conclusions

We developed the GZO-graded ITO cathodes with an EEL by using a RF/DC-graded sputtering for PSCs with enhanced PCE. Compared to the ITO monolayer cathode, the PCE of PSCs based on GZO-graded ITO cathode is raised to 9.67% from 5.25%. The effects of GZO-graded ITO cathodes as a function of the GZO-graded region thickness are systemically investigated in terms of structural, surface morphological, electrical, and optical properties. The performance of the device is sensitive to the thickness of GZO-graded interlayer. Under optimized condition, the GZO-graded ITO cathodes indicate with the sheet resistance of 15.6 Ohm/square, the resistivity of 2.3 × 10^−4^ ohm cm, and the optical transmittance of 95%. Besides, the GZO-graded films showed the well-matched work function of 4.23 eV at the interfaces from the PCBM layer (4.0 eV) and ITO cathode (4.34 eV). Due to the excellent optical, electrical conductivity, and well-matched work function, the PCE of PSCs with GZO-graded ITO cathode exhibited higher than that the PCE of PSCs with ITO monolayer layer and GZO/ITO double layer cathodes. Through the experimental analysis of GZO-graded ITO cathodes, the electron extraction is significantly improved and the charge recombination is suppressed effectively to improve the solar cell performance. This work suggests that the enhanced device operation of PSCs on GZO-graded ITO cathodes by using a graded sputtering indicated very promising integrated electrodes technique, especially in large-scale production.
