# Supplementary material for: ZnO:Ga-graded ITO electrodes to control interface between PCBM and ITO in planar perovskite solar cells
Source: Sci Technol Adv Mater. 2019 Apr 25;20(1):389–400. doi: 10.1080/14686996.2019.1599695 (PMC6493300; doi:10.1080/14686996.2019.1599695)
Supplement: Supplemental Material [file TSTA_A_1599695_SM1862.docx]

**Supplementary information**

**ZnO:Ga graded ITO electrodes to control interface between PCBM and ITO in planar perovskite solar cells**

Hae-Jun Seok^a^, Azmat Ali^b^, Jung-Hwa Seo^b^, Hyun Hwi Lee^c^, Na-Eun Jung^d^, Yeon-jin Yi^d^ and Han-Ki Kim^a,^*

^a^School of Advanced Materials Science and Engineering, Sungkyunkwan University, Suwon, Gyeonggi-do, 16419, Republic of Korea

^b^Department of Materials Physics, Dong-A university, Busan, Republic of Korea

^c^Pohang Accelerator Laboratory, POSTECH, Pohang, Kyungbuk 790-784, Republic of Korea

^d^Institute of Physics and Applied Physics, Yonsei University, Seoul, Republic of Korea

[*E-mail:*](mailto:E-mail:%20imdlhkkim@khu.ac.kr) *hankikim@skku.edu* (Prof. H.-K. Kim) *Tel: +82-31-290-7391 Fax: +82-31-290-7410;*

**
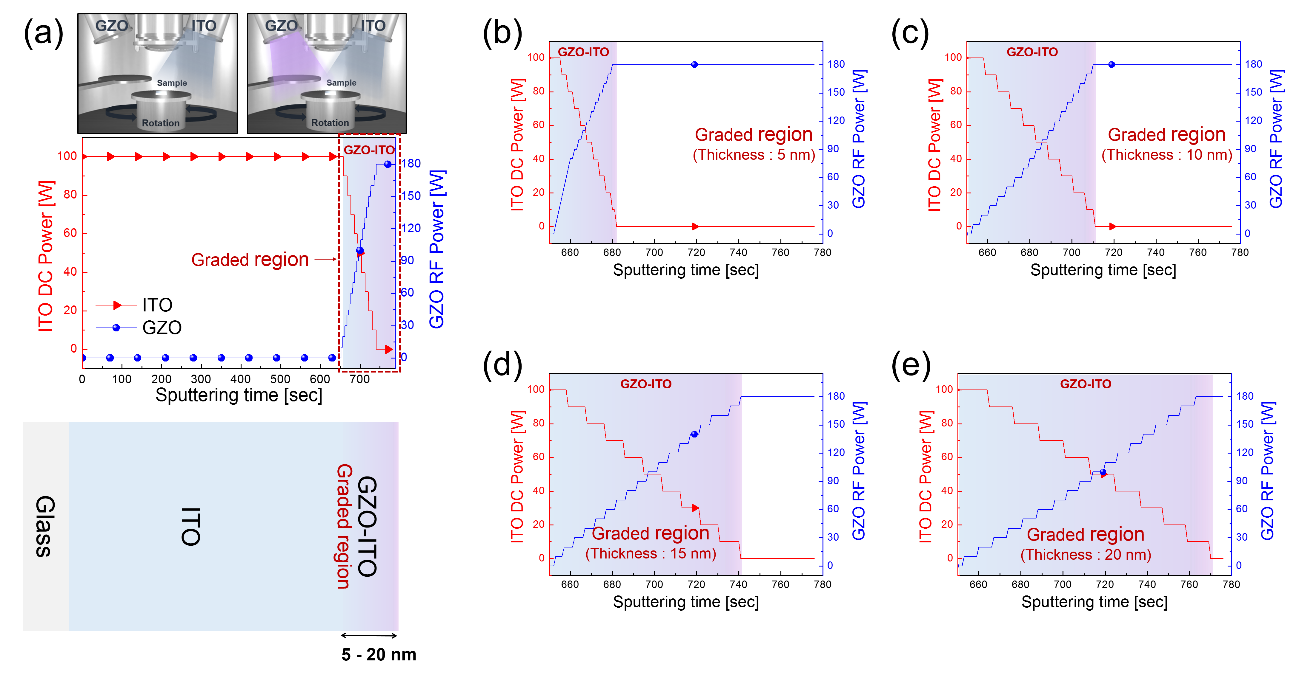
**

**Figure 1S.** (a) Power profile applied to ITO and GZO target during graded co-sputtering as a function of sputtering time and cross-sectional structure of GZO-graded ITO electrode. Top panels demonstrate DC and RF power applied to ITO and GZO target to fabricate GZO-graded ITO electrode for planar n-i-p PSCs. Power profile to prepare (b) 5 nm (29 s), (c) 10 nm (58 s), (d) 15nm (88 s), and (e) 20 nm (117 s) thick Ga:ZnO-ITO graded region.

**
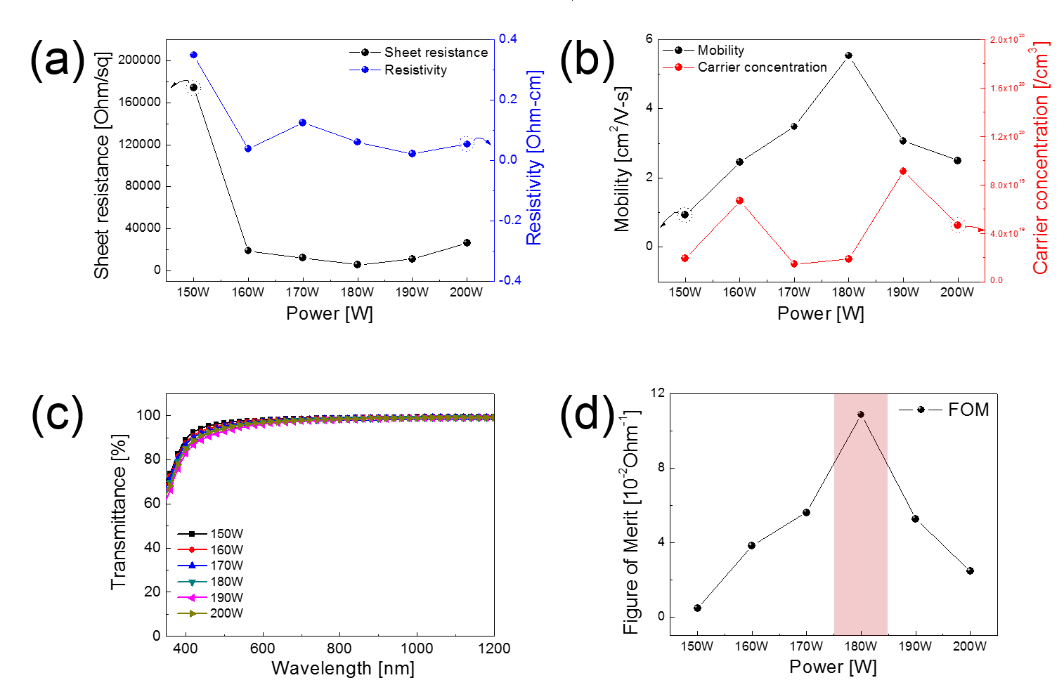
**

**Fig. S2.** (a) Sheet resistance, resistivity, (b) carrier mobility, and concentration, (c) Optical transmittance, (d) FoM value of the GZO single layer as a function of RF power applied to GZO target.


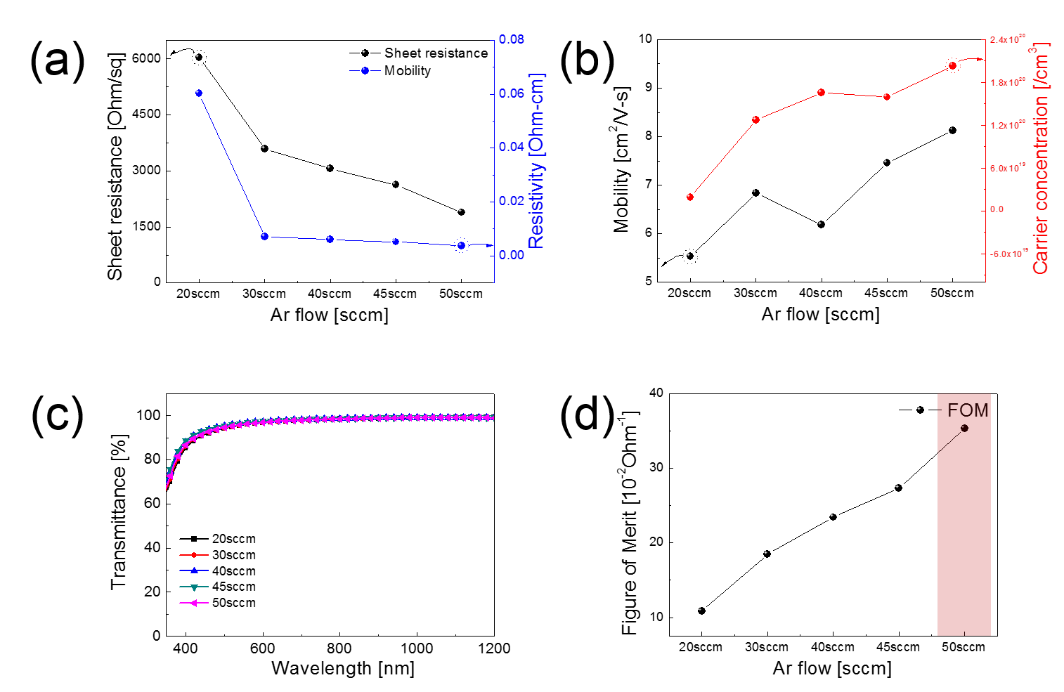


**Fig. S3.** (a) Sheet resistance, resistivity, (b) carrier mobility, and concentration, (c) Optical transmittance, (d) FoM value of the GZO single layer as a function of Ar flow rate.


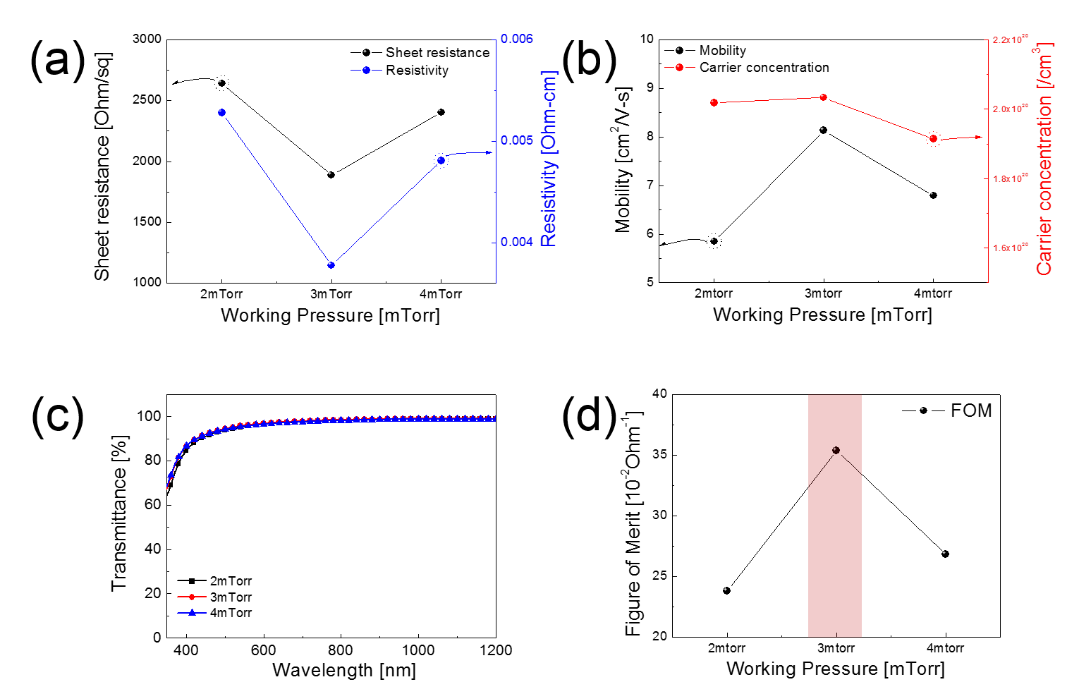


**Fig. S4**. (a) Sheet resistance, resistivity, (b) carrier mobility, and concentration, (c) Optical transmittance, (d) FoM value of the GZO single layer as a function of working pressure.


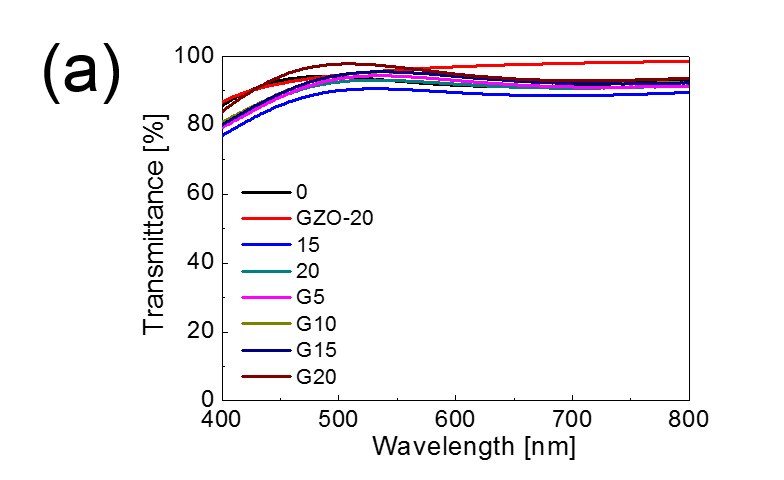


**Fig. S5**. (a) Optical transmittance of ITO monolayer, GZO single layer, GZO/ITO bilayer, and GZO graded ITO electrodes.


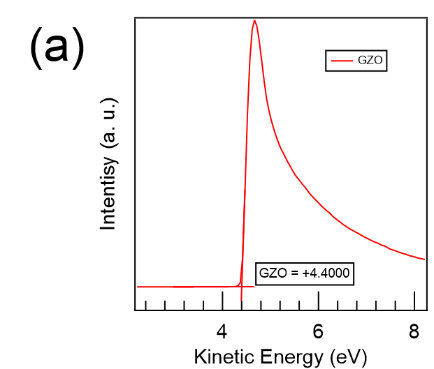


**Fig. S6**. (a) UPS spectrum used to determine work function of GZO in the GZO/ITO bilayer.
